# Association between triglyceride-glucose index and all-cause mortality in non-diabetic patients with fibrotic lung disease receiving antifibrotic therapy: A retrospective cohort study

**DOI:** 10.1371/journal.pone.0357706

**Published:** 2026-09-02

**Authors:** Aysegul Erinc, Ali Kirac, Fatma Ezgi Altun Acar, Abdullah Cagri Onal, Erdogan Cetinkaya

**Affiliations:** Yedikule Chest Diseases and Thoracic Surgery Training and Research Hospital, Health Sciences University, Department of Pulmonology, Istanbul, Turkey; Northwestern University Feinberg School of Medicine, UNITED STATES OF AMERICA

## Abstract

**Introduction:**

Fibrotic lung diseases are associated with significant morbidity and mortality, with a rising prevalence. Prognostic indicators for these diseases include thoracic computed tomography (CT) findings and pulmonary function test (PFT) measures. The triglyceride-glucose (TyG) index, a fasting triglyceride- and glucose-derived surrogate marker of insulin resistance, has been associated with several clinical outcomes; however, its relationship with pulmonary function and all-cause mortality within the non-diabetic range in patients with fibrotic lung disease remains inadequately studied. This study aimed to investigate the association between variability in the TyG index within the non-diabetic range, baseline pulmonary function measures, and all-cause mortality in patients with fibrotic lung disease receiving antifibrotic therapy.

**Methods:**

This retrospective cohort study was conducted on 144 patients with fibrotic lung disease who received antifibrotic therapy between 1 January 2015 and 1 June 2023. Demographic data, comorbidities, smoking status, PFT measures, fasting glucose, triglyceride levels, and survival data were collected. The TyG index was calculated using the formula: Ln [(fasting triglycerides (mg/dL) × fasting glucose (mg/dL)) / 2]. Survival time was calculated from initiation of antifibrotic therapy to death from any cause or last clinical follow-up, with a median follow-up duration of 33.8 months.

**Results:**

The mean age of the cohort was 67.4 years, and 31.9% of the patients were female. The mean TyG index was 8.78. During follow-up, 54 of 144 patients (37.5%) died. In the multivariable Cox regression analysis, the TyG index was not significantly associated with all-cause mortality (adjusted HR = 0.80, 95% CI: 0.40–1.60, p = 0.54). Lower FVC% and greater cumulative smoking exposure were associated with all-cause mortality in the adjusted model.

**Conclusions:**

In this selected cohort of non-diabetic patients with fibrotic lung disease receiving antifibrotic therapy, the TyG index was not significantly associated with all-cause mortality. No consistent association with baseline pulmonary function measures was observed, apart from a weak exploratory inverse correlation with DLCO. However, the restricted metabolic variability and limited statistical precision of the cohort may have reduced the ability to detect modest but potentially clinically meaningful associations. Further prospective studies are needed to clarify the potential prognostic relevance of the TyG index in broader fibrotic lung disease populations.

## Introduction

Fibrotic lung diseases are parenchymal lung disorders characterized by high mortality and morbidity, with an increasing prevalence [1]. The key prognostic indicators for these diseases include thoracic computed tomography imaging findings and pulmonary function test (PFT) measures [1]. Recently, the role of metabolism in chronic lung diseases has become a prominent area of interest [2]. The relationship between metabolic dysfunction, metabolic syndrome, and chronic lung disease has been increasingly explored [3]. The triglyceride-glucose (TyG) index is calculated from fasting triglyceride and glucose concentrations and is widely used as a surrogate marker of insulin resistance. Although insulin resistance, diabetes mellitus, and metabolic syndrome are biologically related, they represent distinct clinical and pathophysiological constructs. The TyG index reflects the fasting triglyceride-glucose component of metabolic status and should not be interpreted as a measure of the entire metabolic syndrome phenotype [4,5]. Although the TyG index has been associated with cardiovascular disease and the development of new-onset diabetes, its relationship with lung health remains inadequately studied [6–8].

Some studies have shown a cross-sectional association between the TyG index and respiratory symptoms, chronic bronchitis, and restrictive-type patterns of pulmonary dysfunction [3]. It has also been reported that metabolic syndrome, IR, and systemic inflammation are important risk markers for reduced lung function in non-smoker healthy individuals [9]. Metabolic syndrome (MetS) is characterized by the clustering of central adiposity, dysglycemia or insulin resistance, hypertension, and atherogenic dyslipidemia and has been associated with chronic lung diseases such as chronic obstructive pulmonary disease (COPD), asthma, and pulmonary fibrosis. Diabetes mellitus may coexist with MetS, but is not required for its diagnosis. Diabetes and hyperglycemia lead to a weakened immune response and increased susceptibility to infections, thus raising the risk of lung infections. Additionally, it has been suggested that increased parasympathetic signal changes associated with high insulin levels may trigger bronchial activity, creating a predisposition to subepithelial fibrosis [10–13]. There are also experimental studies showing that disturbances in carbohydrate and lipid metabolism can lead to changes in surfactant composition [14]. Therefore, elevations in the TyG index may reflect metabolic disturbances related to dyslipidemia and impaired glucose metabolism, both of which have been associated with structural and functional lung changes in translational and experimental studies [3]. However, the association of the TyG index with baseline pulmonary function and all-cause mortality has not been adequately investigated in patients with established fibrotic lung disease receiving antifibrotic therapy.

Insulin resistance has been implicated in fibrotic processes through several proposed mechanisms, including insulin-like growth factor signaling, TGF-β activation, oxidative stress, and metabolic alterations in fibroblasts. On this basis, metabolic indices reflecting insulin resistance, such as the TyG index, have been proposed as potential markers of disease progression in chronic lung diseases [2,15]. However, evidence regarding the relationship of these metabolic indices with longitudinal progression of fibrotic lung disease remains limited.

Therefore, this study aimed to investigate the association between the TyG index, PFT measures, and survival in patients with fibrotic lung disease receiving antifibrotic therapy. Our objective was not to evaluate the effects of established diabetes mellitus, but rather to examine metabolic variability within a non-diabetic population using TyG index as a marker of subclinical insulin resistance. Accordingly, the findings should not be interpreted as reflecting the full spectrum of metabolic dysfunction in fibrotic lung disease populations.

## Materials and methods

### Study design and setting

This retrospective cohort study was conducted at Yedikule Chest Diseases and Thoracic Surgery Training and Research Hospital, a tertiary referral hospital specializing in respiratory diseases. The institution evaluates more than 200 patients with pulmonary fibrosis annually. Patients referred for evaluation of fibrotic lung disease undergo clinical assessment, pulmonary function testing, and high-resolution computed tomography. Decisions regarding the initiation of antifibrotic therapy are made through the institutional Interstitial Lung Diseases Council, consisting of pulmonology, occupational medicine, and radiology specialists. Although the individual council members may have changed during the 2015–2023 study period, antifibrotic treatment decisions were made within this institutional multidisciplinary framework throughout the study period.

### Ethics approval and informed consent statement

This study was carried out in compliance with the Declaration of Helsinki. The Yedikule Chest Diseases and Thoracic Surgery Training and Research Hospital Ethics Committee approved the study protocol (ethical approval date, number: 13 July 2023, 2023−362). The requirement for written informed consent was waived due to the retrospective design. All extracted patient data were de-identified prior to statistical analysis.

### Study population

**Fig 1:**
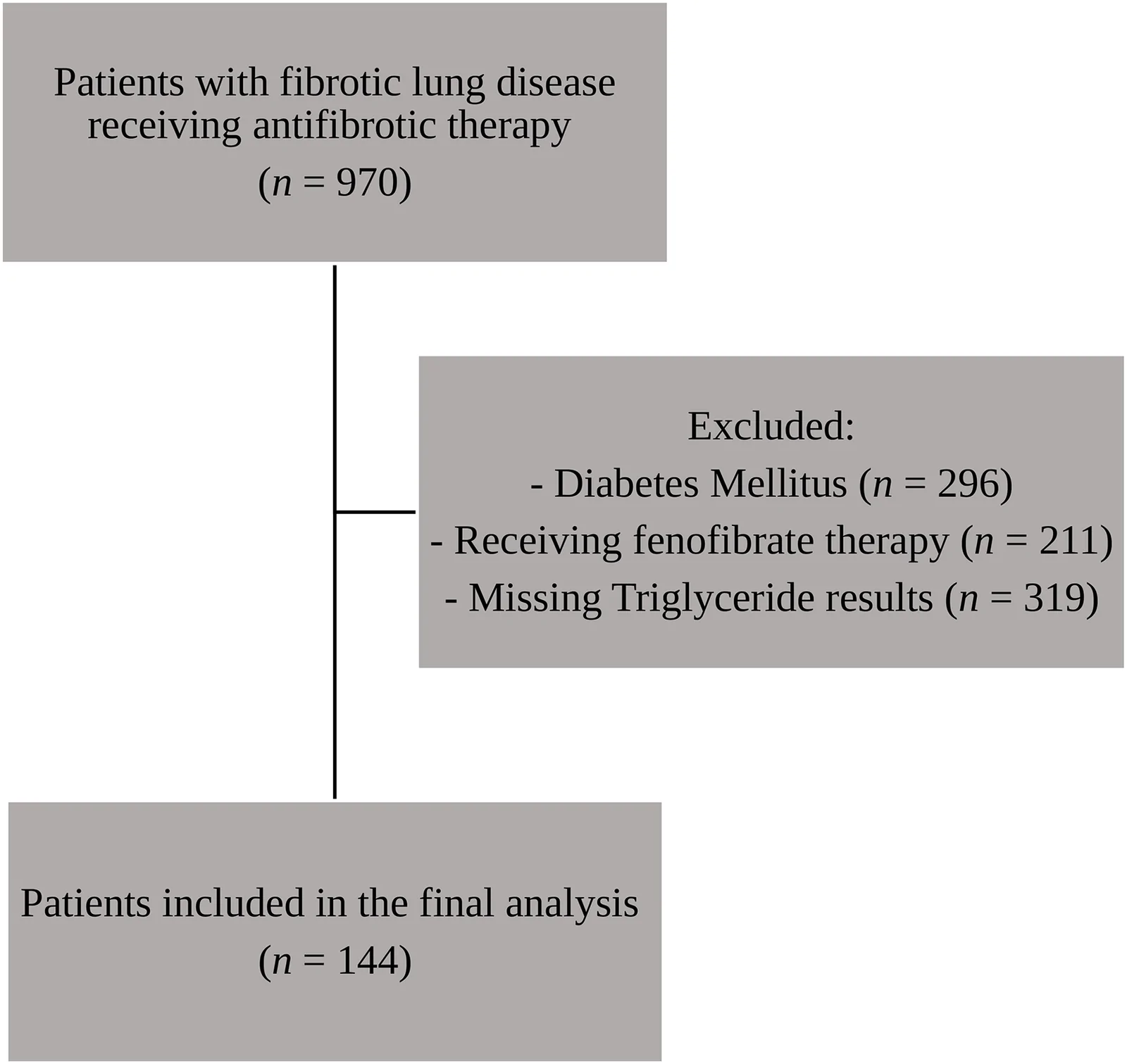
Flowchart of the study population.

This retrospective cohort study included adult patients (≥18 years) with fibrotic lung disease who received antifibrotic therapy at our center between 1 January 2015 and 1 June 2023. A total of 970 patients were initially screened for eligibility. Patients with a documented diagnosis of diabetes mellitus, those receiving fenofibrate therapy, and those without available triglyceride measurements were excluded to minimize pharmacological and disease-related influences on glucose and triglyceride levels and to allow evaluation of the TyG index as a marker of subclinical metabolic dysfunction within the non-diabetic range. After application of all predefined inclusion and exclusion criteria, 144 patients constituted the final analytic cohort. Fibrotic lung disease was defined based on radiological evidence of fibrosis on high-resolution computed tomography, including honeycombing, traction bronchiectasis or bronchiolectasis, and interlobular septal thickening. Interlobular septal thickening alone was not considered sufficient for inclusion and was required to be present as part of an overall radiologic pattern compatible with fibrotic lung disease. Because standardized retrospective classification into specific interstitial lung disease subtypes was not uniformly available, the study cohort represented a clinically heterogeneous group of fibrotic lung diseases rather than a single homogeneous diagnostic entity. The study flowchart is presented in Fig 1.

### Data collection

All data used in this study were accessed and extracted from the hospital’s electronic medical record system between 15 July 2023 and 20 October 2023. Demographic characteristics and comorbidities, including hypertension, asthma, chronic obstructive pulmonary disease, cardiovascular disease, and malignancy, were identified retrospectively from physician-documented diagnoses in the electronic medical records. No independent retrospective adjudication or uniform study-specific diagnostic criteria were applied for these comorbidities. Smoking status and cumulative smoking exposure in pack-years were recorded.

Lung function test parameters, including forced vital capacity (FVC) (liters), FVC (%), diffusing capacity of the lung for carbon monoxide (DLCO) (mL/min/mmHg), and DLCO (%), as well as fasting blood glucose and triglyceride measurements routinely obtained as part of baseline clinical evaluation, and the 6-minute walk test (6-MWT) results were recorded from the hospital system. The forced expiratory volume in 1 second (FEV₁)%pred / FVC%pred value was calculated as (FEV₁ percent predicted / FVC percent predicted) × 100 and does not represent the conventional raw FEV₁/FVC ratio. Baseline fasting glucose and triglyceride measurements used for calculation of the TyG index were obtained at the time of antifibrotic therapy initiation or within a predefined one-month time window, and the closest available values were used. Fasting status was not independently verifiable for every laboratory measurement and was determined based on the recorded laboratory designation and routine institutional practice of obtaining baseline glucose and triglyceride measurements after an overnight fast. All functional parameters (FVC, DLCO, and 6-MWT) were obtained at baseline, corresponding to the date closest to the initiation of antifibrotic therapy. Only baseline measurements were used in the correlation and survival analyses. The death status of the patients was recorded, and the dates of antifibrotic treatment initiation and death were obtained from the system.

### Definitions

Diabetes mellitus was defined based on a physician-documented diagnosis recorded in the electronic medical record. Fasting glucose or HbA1c values were not used retrospectively to establish a new diagnosis of diabetes in patients without a documented clinical diagnosis. Smoking status was categorized as follows: current smokers were those who had smoked at least 100 cigarettes and reported smoking daily; former smokers were individuals with a lifetime history of smoking but who were not currently smoking; and never smokers were those who had smoked fewer than 100 cigarettes in their lifetime [16]. Patients exhibiting honeycombing, traction bronchiectasis/bronchiolectasis, or interlobular septal thickening within the lung parenchyma on thoracic computed tomography images were classified as having fibrotic lung disease. At our institution, the initiation of antifibrotic therapy, including pirfenidone or nintedanib, for patients is determined by the Interstitial Lung Diseases Council, which consists of a pulmonologist, an occupational medicine specialist, and a radiologist. The TyG index was calculated using the formula: Ln [(fasting triglycerides (mg/dL) × fasting glucose (mg/dL)) / 2] **[**5**].**

### Outcomes

The primary outcome of the study was all-cause mortality, defined as death from any cause during the follow-up period. The primary objective was to assess the association between the TyG index and survival in patients receiving antifibrotic treatment. Secondary outcomes were defined to further explore the clinical relevance of metabolic dysfunction in this population. These included evaluating the association between the TyG index and baseline pulmonary function parameters (FVC, FEV₁ and DLCO), examining the relationship between the TyG index and functional capacity measured by the (6-MWT), and comparing metabolic parameters (fasting glucose, triglycerides, and TyG) between deceased and surviving patients. Additionally, correlations between the TyG index and pulmonary function measures were analyzed to determine whether the TyG index demonstrated any measurable link to baseline respiratory impairment.

### Data analysis and statistical methods

Because this was a retrospective cohort study, no a priori sample-size calculation was performed. All patients who met the eligibility criteria during the predefined study period were included in the analysis. We used descriptive statistics to present the data. Categorical variables were presented as numbers and percentages. The normality of continuous variables was assessed using the Shapiro–Wilk test and visual inspection of histograms. Normally distributed continuous variables were presented as mean ± standard deviation, whereas non-normally distributed variables were presented as median and interquartile range (IQR). Correlations of the TyG index with pulmonary function parameters and the 6-MWT, as well as correlations among functional variables, were assessed using Pearson’s correlation coefficient when both variables were normally distributed and Spearman’s rank correlation coefficient when at least one variable was non-normally distributed. Univariable Cox regression analyses were performed for candidate predictors. Given the limited number of mortality events, the multivariable model was restricted to five variables selected according to the primary study objective, clinical relevance, univariable findings, and multicollinearity considerations. The final model included sex, smoking pack-years, COPD, FVC%, and the TyG index. In the Cox proportional hazards models, continuous variables were entered as continuous terms. Hazard ratios represent a one-pack-year increase in smoking exposure, a one-percentage-point increase in FVC%, and a 50-meter increase in 6-MWT distance; other continuous variables were analyzed in their original measurement units. Categorical variables included in the Cox models were coded using indicator variables, with female sex as the reference category. Smoking status was presented descriptively, whereas cumulative smoking exposure was evaluated using smoking pack-years. Never smokers were assigned zero pack-years in the Cox regression analyses, whereas the descriptive pack-year values were summarized among ever-smokers. Hazard ratios are presented with 95% confidence intervals. For survival analyses, time zero was defined as the date of initiation of antifibrotic therapy. All included patients were incident antifibrotic users, and the recorded treatment-initiation date represented the first initiation of pirfenidone or nintedanib. No patient had documented antifibrotic exposure before cohort entry. Patients entered the cohort at the time of antifibrotic therapy initiation. Survival time was calculated from this date to death from any cause or last available clinical follow-up. Patients who were alive at the end of follow-up were censored at the date of their last documented clinical contact. The proportional hazards assumption was assessed graphically using log-minus-log survival plots, and no clear violations were observed on visual inspection. Formal residual-based testing, such as assessment of Schoenfeld residuals, was not performed. All statistical analyses were performed using IBM SPSS Statistics version 23 and were conducted using a complete-case approach, with no imputation methods applied. P-values less than 0.05 were considered statistically significant.

## Results

This study included a total of 144 patients with fibrotic lung disease who were receiving antifibrotic treatment. Of these patients, 32% were female, and the mean age was 67.4 years. Current smokers constituted 59.7% of the cohort, former smokers 8.3%, and never smokers 25.7%. Among smokers, the median pack-years of smoking was 35 (IQR: 20–45). Comorbidities were present in 69% of the patients, with hypertension (51%), cardiovascular disease (35%), and COPD (24%) being the most common (Table 1). Among the study population, 65 patients (45.1%) received pirfenidone and 79 patients (54.9%) received nintedanib therapy. The median follow-up duration was 33.8 months (IQR: 17.0–55.0 months).

**Table 1:** Baseline Characteristics of the Study Population.

Variables	Total Patients (*n* = 144)	Death (*n* = 54)	Alive (*n* = 90)
Age, years*	67.4 ± 8.7	67.9 ± 9	67.1 ± 8.6
Female sex, *n* (%)	46 (31.9)	15 (27.8)	31 (34.4)
Smoking group, *n* (%)			
- Never smoker	37 (25.7)	14 (25.9)	23 (25.6)
- Former smoker	12 (8.3)	2 (3.7)	10 (11.1)
- Current smoker	86 (59.7)	33 (61.1)	53 (58.9)
- Smoking pack-years †	35 (20-45)	40 (20-50)	30 (19-45)
Comorbidities, *n* (%)			
- At least one comorbidity	99 (68.8)	39 (72.2)	60 (66.7)
- Hypertension	74 (51.4)	26 (48.1)	48 (53.3)
- Asthma	14 (9.7)	4 (7.4)	10 (11.1)
- COPD	35 (24.3)	18 (33.3)	17 (18.9)
- CVD	50 (34.7)	22 (40.7)	28 (31.1)
- Malignancy	16 (11.1)	6 (11.1)	10 (11.1)
PFT measures			
- FEV1, L †	2.12 (1.65-2.56)	2.02 (1.51-2.4)	2.16 (1.75-2.7)
- FEV1, % *	79.2 ± 20.6	76 ± 20.5	81 ± 20.6
- FVC, L *	2.51 ± 0.8	2.35 ± 0.75	2.61 ± 0.82
- FVC, % *	74.2 ± 19.1	70.8 ± 19.4	76.2 ± 18.6
- FEV1%pred / FVC%pred, % †	91 (82-112)	89 (83-115)	93 (82-110)
- DLCO, mL/min/mmHg †	8 (4.7-12)	4.83 (3.26-7.6)	9.9 (6.6-12.7)
- DLCO, % *	50.6 ± 19.5	45 ± 18.2	53.6 ± 19.6
6-MWT, meter*	384 ± 131	336 ± 139	410 ± 119
Triglyceride, mg/dL †	123 (92-169)	124 (91-156)	123 (92-180)
Glucose, mg/dL †	100 (91-110)	104 (92-121)	98 (90-108)
TyG*	8.78 ± 0.52	8.77 ± 0.49	8.79 ± 0.53

COPD: chronic obstructive pulmonary disease; CVD: cardiovascular disease; PFT: pulmonary function tests; FEV1: forced expiratory volume in 1 second; FVC: forced vital capacity; DLCO: diffusing capacity of the lung for carbon monoxide; 6-MWT: 6-minute walk test; TyG: triglyceride-glucose index. *Parameters that follow a normal distribution are presented as mean ± standard deviation. † Parameters that did not follow a normal distribution are presented as median and interquartile range. Smoking status data were unavailable for 9 patients (5 deceased and 4 surviving); therefore, the smoking-status categories do not sum to the total cohort.

PFT results showed a mean FEV₁% of 79%, a mean FVC% of 74.2%, and a mean DLCO% of 50.6%. The mean distance covered in the 6-MWT was 384 meters. Regarding metabolic parameters, the median triglyceride level was 123 mg/dL (IQR: 92–169), and the median glucose level was 100 mg/dL (IQR: 91–110). The mean TyG was calculated as 8.78. During the follow-up period, 54 patients (37.5%) died.

In exploratory correlation analyses, no statistically significant association was observed between the TyG index and most baseline pulmonary function measures. A weak inverse correlation was observed between the TyG index and DLCO (Spearman’s ρ = −0.21, p = 0.02). Because multiple correlations were examined without adjustment for multiplicity, this isolated finding should be interpreted cautiously and may represent a chance association.

In the univariate Cox regression analysis evaluating associations with all-cause mortality, no statistically significant associations were observed for age, sex, hypertension, asthma, cardiovascular disease, or malignancy. Similarly, pulmonary function parameters, including FEV₁ (L), FEV₁%, FVC (L), and the FEV₁/FVC ratio, were comparable between the two groups (p > 0.05) (Table 2).

**Table 2:** Univariate and Multivariable Cox Regression.

Univariate Cox Regression				Multivariable Cox Regression		
Variable	Hazard ratio	CI (95%)	*p*	Hazard ratio	CI (95%)	*p*
Age	1.02	0.98-1.05	0.21			
Male sex	1.72	0.93-3.20	0.08	1.55	0.49–4.89	0.45
Smoking, pack/years	1.01	1.00-1.02	0.03	1.01	1.0–1.02	**0.047**
Comorbidities						
- At least one	1.10	0.60-2.01	0.74			
- Hypertension	0.84	0.48-1.44	0.53			
- Asthma	0.39	0.11-1.32	0.13			
- COPD	2.26	1.27-4.03	0.005	1.65	0.75-3.66	0.21
- CVD	1.43	0.82-2.48	0.20			
- Malignancy	1.42	0.60-3.36	0.41			
PFT parameters						
- FEV1, L	0.79	0.5-1.24	0.30			
- FEV1%	0.98	0.97-1.0	0.14			
- FVC, L	0.80	0.55-1.16	0.24			
- FVC%	0.98	0.96-0.99	0.023	0.97	0.95-0.99	**0.02**
- FEV1%pred / FVC%pred	1.0	0.99-1.02	0.31			
- DLCO, mL/min/mmHg	0.91	0.85-0.93	0.016			
- DLCO, %	0.98	0.96-0.99	0.03			
6-minute walk test	0.82	0.71-0.96	0.011			
Triglyceride	0.99	0.99-1.03	0.67			
Glucose	1.0	0.99-1.0	0.88			
TyG	1.0	0.60-1.66	0.97	0.80	0.40-1.60	0.54

CI: confidence interval; COPD: chronic obstructive pulmonary disease; CVD: cardiovascular disease; PFT: pulmonary function tests; FEV1: forced expiratory volume in 1 second; FVC: forced vital capacity; DLCO: diffusing capacity of the lung for carbon monoxide; TyG: triglyceride-glucose index.

In the univariable Cox regression analyses, greater smoking exposure and the presence of COPD were associated with higher all-cause mortality (p = 0.03 and p = 0.005, respectively). Higher FVC%, DLCO%, DLCO measured in mL/min/mmHg, and 6-MWT distance were associated with lower all-cause mortality (p = 0.023, p = 0.03, p = 0.016, and p = 0.011, respectively).

Triglyceride, glucose, and the TyG index were not significantly associated with all-cause mortality in the univariable Cox regression analyses (p = 0.67, p = 0.88, and p = 0.97, respectively).

Strong pairwise correlations were observed among FVC%, FEV₁, DLCO, and 6-MWT distance (all correlation coefficients >0.7). Given these correlations and the limited number of mortality events, FVC% was selected as the principal pulmonary function variable to reduce model redundancy and the risk of overfitting. Although asthma showed a trend towards significance (p < 0.15), it was not included in the multivariable Cox regression analysis due to the small number of patients in this subgroup, which may have influenced the observed difference.

Sex, smoking pack-years, presence of COPD, FVC%, and the TyG index, which were considered clinically significant, were included in the multivariable Cox regression analysis. Male sex (HR = 1.55, 95% CI: 0.49–4.89, p = 0.45) and the presence of COPD (HR = 1.65, 95% CI: 0.75–3.66, p = 0.21) were associated with an increased risk of mortality; however, these associations were not statistically significant. Higher smoking pack-years showed a borderline association with all-cause mortality in the adjusted model (HR = 1.01, 95% CI 1.00–1.02, p = 0.047), and this finding should be interpreted cautiously. The TyG index was not significantly associated with mortality in the multivariable analysis (HR = 0.80, 95% CI: 0.40–1.60, p = 0.54), although the wide confidence interval indicates limited precision of the estimate (Table 2).

## Discussion

This study investigated the association between the TyG index and survival in patients with fibrotic lung disease receiving antifibrotic treatment. To the best of our knowledge, this is among the first studies examining the relationship of this index in individuals with fibrotic lung disease. Although previous studies have reported an association between the TyG index and restrictive-type ventilatory patterns in general population-based cohorts, none have specifically evaluated this relationship in patients with established fibrotic lung disease. Therefore, our study is among the first to investigate the prognostic relevance of TyG index in this distinct clinical population. Our results indicate that TyG index was not significantly associated with mortality in this patient cohort. In contrast, lower FVC% and greater cumulative smoking exposure were associated with all-cause mortality in the adjusted model, although these findings should be interpreted cautiously because several clinically relevant prognostic variables were unavailable for inclusion. These findings are consistent with the established prognostic relevance of pulmonary function impairment and cumulative smoking exposure; however, the present study was not designed to compare the predictive performance of pulmonary and metabolic variables. Accordingly, interpretation of the present findings should be limited to a selected non-diabetic cohort receiving antifibrotic therapy.

Our findings contrast with previous studies that have suggested a link between metabolic dysfunction and pulmonary health. Prior research has shown that metabolic syndrome and insulin resistance are associated with reduced lung function, even in non-smokers [3,9]. However, these studies were conducted in broader populations, including individuals without established lung fibrosis. Our study focused exclusively on patients with fibrotic lung disease, a biologically complex condition involving fibro-proliferative pathways, systemic inflammation, immune dysregulation, oxidative stress, aging-related mechanisms, and comorbidity burden [17]. Previous studies have suggested that oxidative stress may play a significant role through pathways involving endothelial dysfunction, pro-inflammatory cytokine production, and mitochondrial damage in individuals with MetS and elevated TyG [18–21]. These mechanisms have been proposed in experimental and observational studies and may theoretically contribute to pulmonary injury and adverse clinical outcomes in fibrotic lung disease [22]. However, in the present study, the TyG index was not significantly associated with baseline pulmonary function measures or all-cause mortality within the restricted non-diabetic range represented in this selected cohort. This finding should not be interpreted as evidence that metabolic dysfunction is unimportant in fibrotic lung disease or that the TyG index lacks prognostic relevance in patients with diabetes mellitus or more pronounced metabolic abnormalities. Given the multiple exploratory correlations performed and the absence of adjustment for multiplicity, the observed inverse correlation between TyG and DLCO should be interpreted cautiously and may represent a chance association.

Because the TyG index is a surrogate marker of insulin resistance, it captures only a limited aspect of metabolic status [4,5]. It does not directly assess adipokines, inflammatory cytokines such as IL-6 and TNF-α, oxidative stress, endothelial dysfunction, or mitochondrial injury [23]. These unmeasured pathways may be relevant to fibrotic lung disease based on prior literature, but their relationship to the present findings cannot be determined from this study [23,24]. Accordingly, the absence of a statistically significant association between the TyG index and all-cause mortality should not be interpreted as evidence regarding these biological mechanisms.

In contrast to TyG, higher smoking pack-years and lower FVC% were significantly associated with increased mortality risk in our study. These findings are consistent with previous studies identifying smoking history and impaired pulmonary function as established prognostic factors in fibrotic lung disease [25]. Smoking contributes to oxidative stress, epithelial injury, and inflammation, all of which accelerate fibrosis progression [26]. The significant association between higher smoking pack-years and mortality in our study underscores the detrimental effects of cumulative smoke exposure, even in patients receiving antifibrotic treatment. Lower FVC% was associated with increased all-cause mortality risk in the adjusted model, consistent with its established prognostic relevance in fibrotic lung disease [27]. This is consistent with previous studies demonstrating that FVC decline is one of the most reliable predictors of disease progression and survival in idiopathic pulmonary fibrosis and other fibrotic lung diseases [28,29]. Recent real-world studies have expanded the evidence regarding outcomes in antifibrotic-treated progressive pulmonary fibrosis cohorts, while contemporary cohort data have further supported the prognostic relevance of 6-MWT measures in fibrotic lung disease [30,31]. Recent literature has also highlighted potential links between diabetes-related metabolic dysfunction and pulmonary fibrosis [32].

Several limitations should be considered when interpreting the findings of this study. The final analytic cohort represented a highly selected subset of the initially screened population because patients with diabetes mellitus, those receiving fenofibrate therapy, and those without available triglyceride measurements were excluded. This selection process may have introduced selection bias and substantially narrowed the distribution of glucose, triglyceride, and TyG index values within the cohort. In particular, patients with more pronounced metabolic dysfunction, in whom the TyG index might have greater biological and prognostic relevance, were underrepresented. The resulting restriction in metabolic variability may have attenuated or otherwise distorted the observed association between the TyG index and all-cause mortality and may have contributed to the statistically nonsignificant finding. Therefore, the results should not be interpreted as evidence that the TyG index lacks prognostic relevance in the broader fibrotic lung disease population and should be considered applicable only to this selected non-diabetic cohort receiving antifibrotic therapy. The retrospective, treatment-based cohort design may have introduced survivor and selection bias because patients had to survive long enough to complete clinical evaluation, initiate antifibrotic therapy, and have the required baseline laboratory measurements available. Consequently, patients with rapidly progressive disease or death before treatment initiation may have been underrepresented. This selection may have attenuated or otherwise distorted the observed associations, including the non-significant association between the TyG index and all-cause mortality. Furthermore, cohort entry was defined by antifibrotic treatment initiation rather than by the onset or diagnosis of fibrotic lung disease. Baseline clinical characteristics and longitudinal follow-up data were not systematically available for excluded patients, precluding a reliable comparison between included and excluded individuals.

Standardized classification of interstitial lung disease subtypes, centralized radiologic review, and uniform multidisciplinary diagnostic adjudication were not available for all patients. This may have resulted in diagnostic misclassification and a clinically heterogeneous cohort comprising fibrotic lung disease phenotypes with different baseline prognoses and treatment trajectories, potentially influencing the observed survival associations. In addition, the absence of standardized adjudication for comorbidity assessment may have introduced diagnostic misclassification.

Several important prognostic variables, including body mass index, baseline oxygen requirement, radiologic disease extent, ILD subtype, disease duration before antifibrotic therapy, antifibrotic dose and subsequent treatment course—including dose reduction, discontinuation, switching, and adherence—and concomitant corticosteroid, immunosuppressive, statin, and other lipid-lowering therapies were not consistently available and therefore could not be incorporated into the multivariable model. The absence of these variables represents a major source of residual confounding because they may be associated with both metabolic markers and survival.

Although DLCO% and 6-MWT distance represent physiological domains distinct from FVC%, sensitivity models substituting these variables for FVC% were not performed because of variable-level missingness, the limited number of mortality events, and concerns regarding model instability. The absence of these sensitivity analyses may have resulted in residual confounding and limited the assessment of prognostic information beyond pulmonary volume impairment. Formal assessment of nonlinear relationships for continuous covariates was not performed. Missing data were handled using a complete-case approach. This approach may have reduced statistical power and introduced selection bias if the probability of having complete data was related to disease severity, comorbidity burden, or follow-up intensity. A reliable variable-level summary of missingness for all clinical and functional parameters could not be retrospectively reconstructed from the source records, which represents an additional reporting limitation. Variability in the timing of laboratory measurements relative to antifibrotic therapy initiation may have introduced measurement error, particularly in the setting of acute illness, hospitalization, corticosteroid exposure, or medication changes.

Serial changes in pulmonary function, radiologic disease progression, and acute exacerbations were not assessed; therefore, no conclusions can be drawn regarding the association between the TyG index and longitudinal progression of fibrotic lung disease. In addition, cause-specific mortality data were unavailable, limiting disease-specific interpretation of the observed survival associations. Finally, the modest sample size, limited number of mortality events, restricted TyG index variability and wide confidence interval around the TyG estimate limited the statistical precision of the study. Therefore, the study may have been underpowered to detect modest but potentially clinically meaningful associations between the TyG index and all-cause mortality. Accordingly, the findings should be interpreted as hypothesis-generating within a selected non-diabetic cohort.

## Conclusions

In this selected cohort of non-diabetic patients with fibrotic lung disease receiving antifibrotic therapy, the TyG index was not significantly associated with all-cause mortality in the adjusted analysis and did not demonstrate a consistent association with baseline pulmonary function measures, although a weak exploratory inverse correlation with DLCO was observed. However, the wide confidence interval, restricted metabolic variability, and limited number of mortality events preclude the exclusion of modest but potentially clinically meaningful associations. Lower FVC% and greater smoking exposure were associated with mortality in the adjusted analysis, although these findings should be interpreted cautiously given the retrospective design, restricted metabolic variability, and potential residual confounding. Therefore, the present findings should be considered exploratory and hypothesis-generating. Further prospective studies involving broader and more metabolically diverse fibrotic lung disease populations are warranted to clarify the potential prognostic relevance of the TyG index.

## Supporting information

S1 File PLOSOne_Human_Subjects_Research_Checklist. (DOCX)

S2 File TyG DATA SET. (XLSX)
